# The Effect of Interferon-γ and Zoledronate Treatment on Alpha-Tricalcium Phosphate/Collagen Sponge-Mediated Bone-Tissue Engineering

**DOI:** 10.3390/ijms161025678

**Published:** 2015-10-26

**Authors:** Peiqi Li, Yoshiya Hashimoto, Yoshitomo Honda, Yoshiyuki Arima, Naoyuki Matsumoto

**Affiliations:** 1 Department of Orthodontics, Graduate School of Dentistry, Osaka Dental University, 8-1 Kuzuha Hanazonocho, Hirakata, Osaka 573-1121, Japan; E-Mails: li-peiqi@cc.osaka-dent.ac.jp (P.L.); arima.dental@gmail.com (Y.A.); naoyuki@cc.osaka-dent.ac.jp (N.M.); 2 Department of Biomaterials, Osaka Dental University, 8-1 Kuzuha Hanazonocho, Hirakata, Osaka 573-1121, Japan; 3 Institute of Dental Research, Osaka Dental University, 8-1 Kuzuha Hanazonocho, Hirakata, Osaka 573-1121, Japan

**Keywords:** bone tissue engineering, α-TCP/CS, inflammatory response, RANKL, TNF-α, osteoclastogenesis, interferon-γ, zoledronate

## Abstract

Inflammatory responses are frequently associated with the expression of inflammatory cytokines and severe osteoclastogenesis, which significantly affect the efficacy of biomaterials. Recent findings have suggested that interferon (IFN)-γ and zoledronate (Zol) are effective inhibitors of osteoclastogenesis. However, little is known regarding the utility of IFN-γ and Zol in bone tissue engineering. In this study, we generated rat models by generating critically sized defects in calvarias implanted with an alpha-tricalcium phosphate/collagen sponge (α-TCP/CS). At four weeks post-implantation, the rats were divided into IFN-γ, Zol, and control (no treatment) groups. Compared with the control group, the IFN-γ and Zol groups showed remarkable attenuation of severe osteoclastogenesis, leading to a significant enhancement in bone mass. Histomorphometric data and mRNA expression patterns in IFN-γ and Zol-injected rats reflected high bone-turnover with increased bone formation, a reduction in osteoclast numbers, and tumor necrosis factor-α expression. Our results demonstrated that the administration of IFN-γ and Zol enhanced bone regeneration of α-TCP/CS implants by enhancing bone formation, while hampering excess bone resorption.

## 1. Introduction

The use of composites of extracellular matrix-like collagen and calcium phosphates as bone fillers for orthopedic and stomatology surgery has increased in recent years, since they promote cell adhesion and differentiation and are degraded under physiological conditions [1]. Results from our previous study found that materials created with porous α-tricalcium phosphate (TCP) and collagen sponges (CS) could be used to treat bone defects. In addition, the resorbable α-TCP/CS implants were replaced with new bone without any side effects [2]. However, α-TCP/CSs are osteoconductive materials that act as space maintainers during bone formation, and their interactions with molecules that stimulate osteogenesis or inhibit osteoclastogenesis are needed to induce bone regeneration in large bone defects.

In general, late-stage immunogenic and inflammatory responses during biomaterial implantations include chronic inflammation, granulation tissue development, foreign body reactions, and fibrosis/fibrous capsule development [3]. The presence of mitogens, chemoattractant cytokines, growth factors, and other bioactive agents generates a rich milieu of activating and inhibitory substances capable of modulating macrophage activity, which lead to severe osteoclastogenesis and affect bone regeneration [4]. The results of several studies have suggested that activated osteoclasts and osteolysis caused by the overexpression of inflammatory cytokines are the main causes of implantation failures [5,6]. Immunogenic responses elicit high levels of inflammatory cytokines involved in osteoclast differentiation, and macrophage infiltration occurs in the fluid and fibrotic tissue surrounding implants [7,8,9]. Inflammatory cytokines such as tumor necrosis factor (TNF)-α or interleukin (IL)-1β function in collaboration with receptor activator of nuclear factor-kappa B ligand (RANKL) and dramatically enhance osteoclast precursor maturation and migration [5,10]. Osteoclasts and associated acute inflammatory reactions promote material degradation and defect repair at the early-recovery stage [11], but severe osteoclastogenesis accompanied with an inflammatory response at the late-recovery stage may significantly inhibit bone regeneration [12].

Zoledronate (Zol) hampers osteoclastic bone resorption and is an important drug for the treatment of various bone diseases. It has previously been shown *in vivo* that the local treatment of β-TCP granules with Zol can increase implant fixation by improving osteogenesis and hampering bone resorption [13]. The beneficial effects of Zol on enhancing osteocyte function, accelerating the generation of new bone, and lowering the bone-resorption activities of osteoclasts and macrophages are clear. Zol treatment during bone-material implantation may serve as an effective modulator of inflammatory responses caused by macrophages, thereby reducing excess inflammatory cytokine expression and osteoclastogenesis.

In addition, interferon (IFN)-γ, a cytokine released by TH1 cells during adaptive immunity, is a proinflammatory cytokine that can cause classically activated macrophages to secrete inflammatory cytokines [14]. However, recent findings have suggested that IFN-γ is a critical regulator of bone resorption and is required for the osteogenic differentiation of mesenchymal stem cells [15,16,17,18]. Although the role of IFN-γ *in vivo* is still controversial [19], IFN-γ may play an important role in bone formation and reducing inflammatory bone resorption *in vivo*, having the potential to enhance material implantation efficacy [17,20].

The local application of IFN-γ or Zol to bone microenvironments may be expected to modulate immunogenic responses, osteogenesis, and osteoclastogenesis. In this study, we showed that IFN-γ and Zol administration can enhance bone formation in a rat calvaria defect model with α-TCP/CS implants, which were generated using a dehydrothermal cross-linking method.

## 2. Results

### 2.1. Material Observations and Measurements

**Figure 1:**
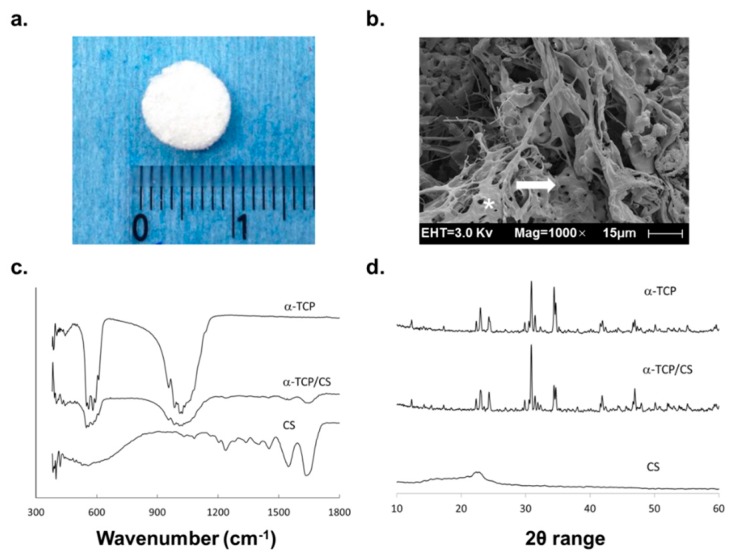
Alpha-tricalcium phosphate (α-TCP)/collagen sponge (CS) material observations and measurements. (**a**) Visual image of an α-TCP/CS; (**b**) SEM image of an α-TCP/CS. The white asterisk is overlaid on a collagen fiber, and the white arrows point to an α-TCP particle; (**c**) FTIR spectra recordings of an α-TCP powder, an α-TCP/CS; and a CS (**d**) XRD patterns of α-TCP/CS particles, α-TCP particles, and a CS.

In SEM images, we observed that collagen fibers were intertwined with α-TCP in the α-TCP/CSs (Figure 1b). Analysis of infrared (IR) spectra results from α-TCP powders and α-TCP/CS materials revealed the presence of α-TCP bands at 1010 and 543 cm^−1^. The bands in the 1180–1140 cm^−1^ range, together with those at 610 and 595 cm^−1^, are attributable to the SO_4_^2−^ group [21]. Characteristic IR spectra were observed for α-TCP/collagen and α-TCP/CS show characteristic IR spectra, with amide-I absorption bands detected at 1650 cm^−1^, amide-II bands detected at 1560 cm^−1^, and a set of three weaker bands representing amide-III vibration modes centered at 1245 cm^−1^ [22] (Figure 1c). Figure 1d shows the X-ray diffraction (XRD) patterns of α-TCP/CS, the original α-TCP particles, and collagen. A comparison of the scatter-plot data of the synthesized α-TCP particles with that of α-TCP data registered with the Joint Committee on Powder Diffraction Standards confirmed that these peaks appeared at the same angles.

### 2.2. Effect of Local IFN-γ and Zol Administration on Bone Turnover after Material Implantation

**Figure 2:**
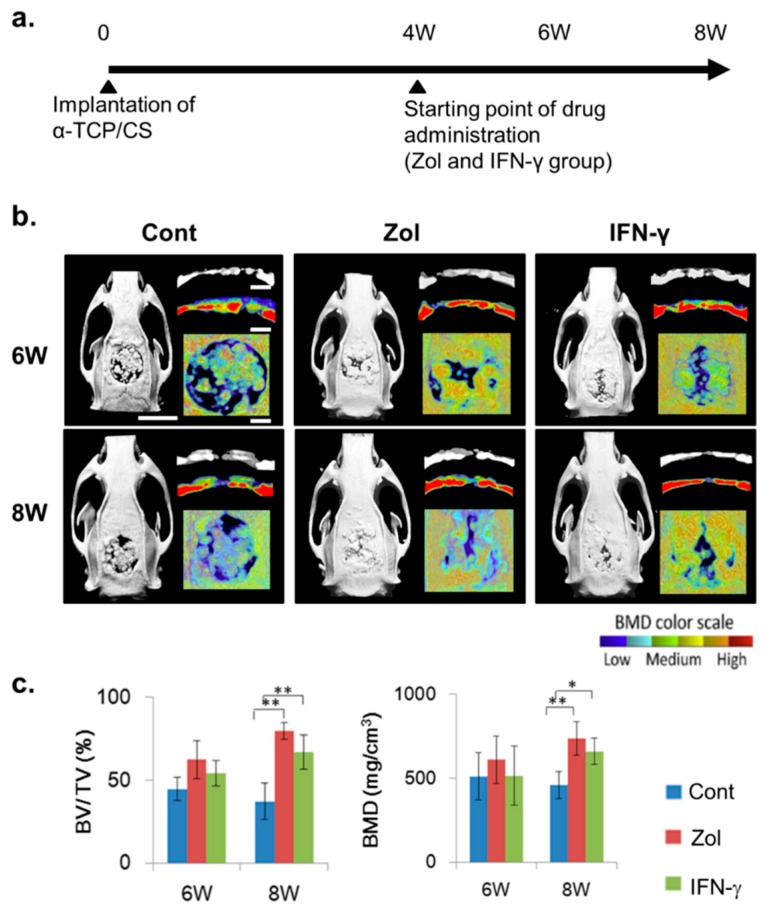
*In vivo* models generated with critically sized defects in rat calvarias filled with α-TCP/CS material. (**a**) After four weeks, rats were administered interferon (IFN)-γ, and bone regeneration was compared with that occurring in no-drug-treated control rats and zoledronate (Zol)-injected rats; (**b**) Micro-computed tomography and bone-mineral density (BMD) images of rat calvarias defects. Scale bars = 10 mm (long bars) or 2 mm (short bars); (**c**) Post-operative bone volumes/tissue volumes (BV/TV) and BMDs were measured each week. The data shown represent the mean ± standard deviation (SD; *n* = 4). * *p* < 0.05, ** *p* < 0.01 (Tukey–Kramer method).

We examined morphometric changes occurring after material-implantation surgery (Figure 2a). In rats not administered IFN-γ or Zol, the micro-computed tomography (CT) images and structural parameters of rat calvaria showed large bone defects at eight weeks following surgery, with a large area of low-density tissue (Figure 2b). The average bone volume in the bone defects decreased from 44.65% (six weeks) to 37.2% (eight weeks), and the bone mineral density (BMD) decreased from 511.9 to 458.6 mg/cm^−3^ (Figure 2c). However, significantly higher bone volumes (79.7%, Zol, *p* < 0.01; 67.1%, IFN-γ, *p* < 0.01) and BMDs (738.3 mg/cm^−3^, Zol, *p* < 0.01; 660.3 mg/cm^−3^, IFN-γ, *p* < 0.05) were observed at Week 8 following Zol or IFN-γ administration at four weeks post-implantation, compared with the control group (Figure 2c).

**Figure 3:**
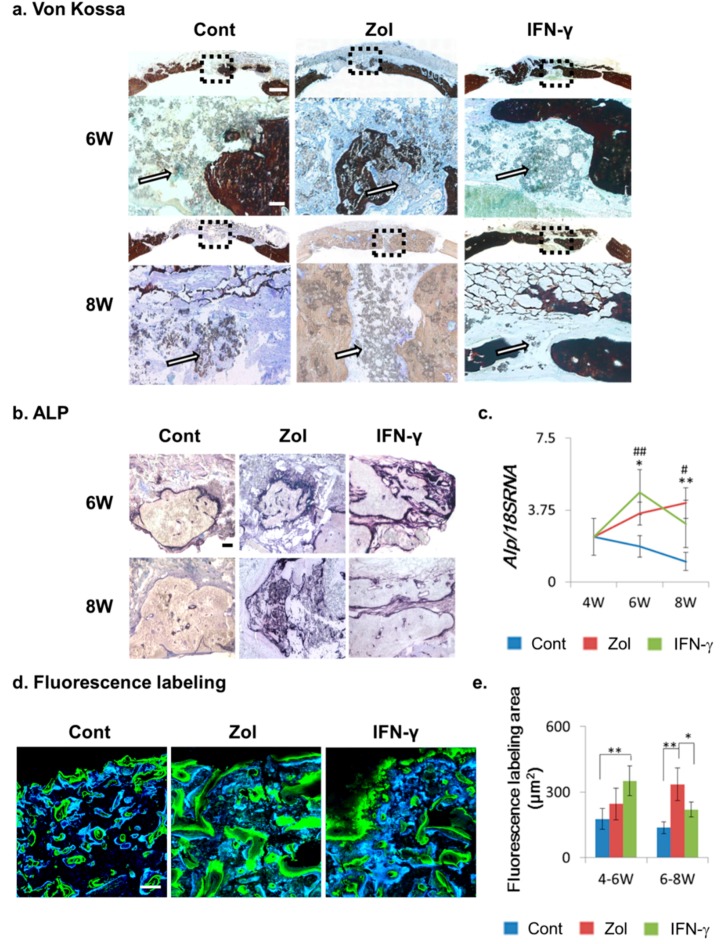
Bone-turnover capacities of material implants following drug treatment. (**a**) Von Kossa staining. Brown staining represents bone tissue, and white arrows show the α-TCP/CS particles in the bone defect. Broken squares represent the magnified areas; (**b**) Alkaline phosphatase (ALP) staining. Black staining represents ALP-positive tissue; (**c**) *Alp* mRNA expression in bone defects. Data show the mean ± SD (*n* = 4). ^#^ Control *vs.* IFN-γ; * Control *vs.* Zol; *^,#^ *p* < 0.05; **^,##^ *p* < 0.01 (Tukey–Kramer method); (**d**) Fluorescence labeling analysis. Calcein (blue staining: new bone growth at 4–6 weeks post-implantation) and tetracycline (green: new bone growth at 6–8 weeks) labeling of regenerative bone tissue in calvarial defects. Scale bars: von Kossa = 1.8 mm and 120 μm (magnified areas), ALP staining = 120 μm, fluorescence labeling = 100 μm; (**e**) Quantification of labeling fluorescence. The data show the mean ± SD (*n* = 4). * *p* < 0.05, ** *p* < 0.01 (Tukey–Kramer method).

We then studied bone regeneration and metabolism using the von Kossa staining method, where the dark brown regions represent the bone tissue (Figure 3a). By von Kossa staining, we found that the amount of bone tissue decreased from Weeks 6–8 in the control group, while clearly distinguishable bone turnover occurred in the Zol- and IFN-γ-injection groups. In addition, von Kossa staining showed that in the eight-week control and Zol groups, considerable α-TCP/CS particles remained in the defect areas (Figure 3a, white arrows). However, in the eight-week IFN-γ-injection group, a reduction of α-TCP/CS particles was observed in the defect areas (Figure 3a, white arrows).

We next studied alkaline phosphatase (ALP) expression using immunohistochemistry to assess the bone turnover capacity in each group (Figure 3b). Weak ALP expression was observed with the six- and eight-week control groups. The six- and eight-week IFN-γ and Zol groups showed clear ALP expression, indicating that the osteoblasts facilitated bone defect repair (Figure 3b). In agreement, the *Alp* mRNA expression level in the Zol and IFN-γ injection groups was significantly increased at six and eight weeks post-implantation, indicating that osteogenesis was increased in the Zol and IFN-γ injection groups, compared to the control group (Figure 3e).

Fluorescence imaging results indicated that a significantly higher mineral deposition rate occurred in the IFN-γ- and Zol -treated defects, compared to that observed in control rats receiving no treatment. In addition, the IFN-γ-treated group showed significantly higher mineral apposition in the 4–6-week group than in the control and Zol groups (Figure 3c,d), whereas the Zol group showed higher mineral apposition during weeks 6–8. These results indicated that increased bone-formation activity occurred after Zol and IFN-γ administration.

### 2.3. Effect of Local IFN-γ and Zol Administration on Osteoclastogenesis and TNF-α and RANKL Expression

**Figure 4:**
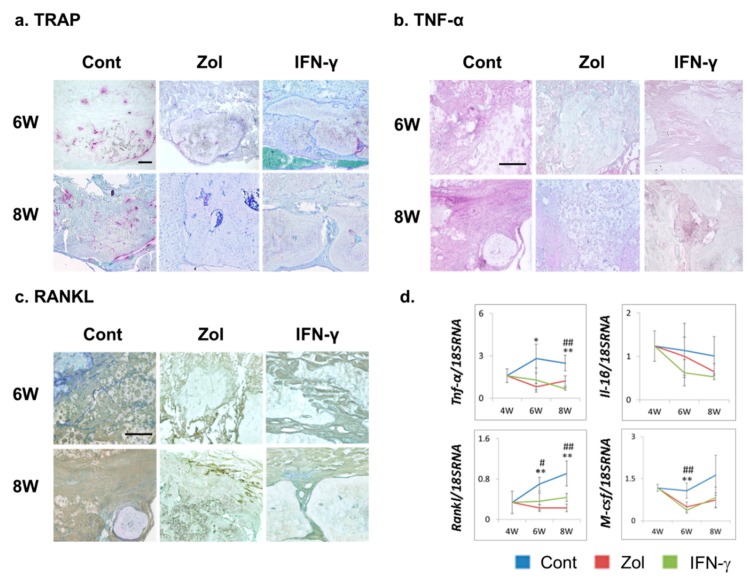
Effect of Zol and IFN-γ administration on osteoclastogenesis and immune responses in defects treated with α-TCP/CS. (**a**) Tartrate-resistant acid phosphatase (TRAP) staining representing the presence of osteoclasts (TRAP-positive cells) in tissue sections; (**b**,**c**) Tumor necrosis factor alpha (TNF-α; purple) and receptor activator of nuclear factor-kappa B ligand (RANKL) expression (brown). Scale bars: 120 μm; (**d**) Expression of genes closely related to osteoclast differentiation and bone resorption (*Rankl*, *Tnf-*α, *Il-1*β, and *M-csf*). The data show the mean ± SD (*n* = 4). ^#^ Control *vs.* IFN-γ, * Control *vs.* Zol; *^,#^ *p* < 0.05, **^,##^ *p* < 0.01 (Tukey–Kramer method).

The expression of tartrate-resistant acid phosphatase (TRAP), a marker of activated osteoclasts, was assessed at each time point. TRAP staining revealed numerous TRAP-positive cells in control-group tissue sections at six or eight weeks post-treatment, while TRAP staining was attenuated in the IFN-γ- and Zol-treated groups (Figure 4a). Inflammatory cytokines such as TNF-α modulate osteoclastogenesis. To investigate the mechanism whereby IFN-γ or Zol treatment suppressed osteoclastogenesis, we further evaluated immune responses in tissues surrounding the implants via immunostaining and quantitative reverse transcriptase-polymerase chain reactions (RT-PCR). Robust TNF-α and RANKL staining were observed at six and eight weeks post-transplantation in the control groups, while Zol and IFN-γ administration markedly reduced TNF-α and RANKL staining (Figure 4b,c). In agreement with the immunostaining results, the mRNA expression levels of the inflammatory cytokines *Tnf-α* and the osteoclastic cytokines *Rankl* and *macrophage colony stimulating factor* (*M-csf*) were significantly lower in tissues treated with IFN-γ and Zol. These results suggested that IFN-γ and Zol treatment both hindered osteoclastogenesis and inflammatory responses surrounding the implants.

## 3. Discussion

Effective bone-regeneration scaffolds should be capable of osteoconduction, osteoinduction, and eventual biodegradation. Moreover, scaffolds for bone regeneration should mimic bone morphology, structure, and function. Natural bones are a complex assembly of type-I collagen nanofibrils and hydroxyapatite (HA). A drawback of using HA in bone-regeneration scaffolds is that it has a low biodegradation rate. The *in vivo*-degradation rate of TCP is higher than that of HA. In addition, α-TCP dissolves more easily in water than does β-TCP, even though they have identical chemical compositions [23]. In the present study, SEM, Fourier-transform infrared (FTIR) spectroscopy, and XRD results demonstrated that α-TCP/CS particles with a three-dimensional porous structure and anatomizing network were created using a dehydrothermal cross-linking method.

In this study, we used a defect size of 9 mm in diameter, which can be considered critical for new bone formation in most defects. Micro-CT imaging showed that the defects were not completely filed with bone at six-weeks following α-TCP/CS implantation. In addition, the BMD and bone-volume values decreased from Week 6 to Week 8. This finding may be explained by evidence that excess post-implantation bone resorption correlates with TNF-α and RANKL over-expression. α-TCP/CS led to upregulated TNF-α and RANKL expression, which resulted in osteoclastogenesis of regenerated bone tissue. TNF-α, a major chronic inflammatory cytokine, was shown to induce rheumatoid arthritis, osteoporosis, periodontal disease, and implantation failures [24]. A large area of TNF-α-RANKL-positive fibrotic tissue surrounding implants or invading regenerated bone was observed, along with numerous activated osteoclasts. Gretzer *et al.* found that TNF-α expression in the exudate was mild at 7 days, but it became significantly higher at 21 days post-implantation, with substantial fibrous repair occurring around the implanted materials [25]. TNF-α can increase the differentiation of RANKL-primed osteoclast precursors, activate bone resorption, and increase osteoclast survival. There is evidence that increased activation and differentiation of osteoclasts by particle-stimulated macrophages may serve important roles, as various findings have shown that supernatants from particle-stimulated macrophages can induce bone resorption in isolated murine calvaria *in vitro* [9].

Immune responses to material implantation trigger osteoclastogenesis and bone destruction. Understanding the relationship between osteoclastogenesis and implantation failure has led to investigations of new routes for therapeutic intervention. However, there is no approved drug therapy to prevent or inhibit material-induced osteoclastogenesis. Zol is used to treat metabolic bone diseases, such as osteoporosis, and inhibits the formation of ruffled borders, trafficking of lysosomal enzymes, and transcytosis of degraded bone matrix, which together block osteoclast functions. Currently, however, there is no evidence in the literature that these drugs can effectively treat implantation failure in patients. In this study, we demonstrated that locally Zol-injected bone defects with α-TCP/CS implantation exhibit significantly higher bone regeneration and decreased inflammatory cytokine expression, leading to a reduction in osteoclastogenesis in new bone formation. These findings indicate the potential of Zol treatment in facilitating successful material-implantation procedures.

Inflammatory macrophage responses are initiated by material particles in bone defects, leading to increased RANKL and TNF-α production in fibrous and stromal cells. In our opinion, treating patients with local immunomodulatory agents that can modulate cytokine expression from macrophages is possible. The effects of RANKL and TNF-α may be down regulated by IFN-γ. IFN-γ is produced locally in the bone microenvironment mainly by cells of immune origin and mesenchymal stem cells, and it plays crucial roles in the regulation of a wide variety of innate and adaptive immune responses [26]. It has been shown that IFN-γ interferes with osteoclast differentiation induced by RANKL and directly inhibits TNF-induced osteoclastogenesis [15]. In addition, IFN-γ is reported to enhance immunosuppressive functions to protect implants against acute rejection [27] and to prevent excess fibrosis during wound healing [28]. In this study, we locally injected IFN-γ at the implantation sites, which led to enhanced osteogenic capacity and reduced bone resorption, suggesting that IFN-γ may also be effective in the treatment of material implants.

## 4. Experimental Section

### 4.1. Biomaterial Preparation

Large and small α-TCP particles were prepared by pulverizing an α-TCP block with a continuous pore structure of 80% pores (Taihei Chemical Industrial Co., Ltd., Osaka, Japan). The median sizes of the large and small particles were 580.8 and 136.2 μm. The particles were mixed at 1:1 mass ratio. Collagen (Nippon Meat Packers, Osaka, Japan) was prepared from a lyophilized powder of pepsin-digested atelocollagen isolated from porcine dermis. α-TCP particles of 150 mg were added to 1 mL homogenized collagen solution. This mixture was poured into plastic molds and immediately frozen to −80 °C and freeze-dried for 24 h. Then, the sponge-like materials underwent dehydrothermal treatment at 140 °C for 24 h in a vacuum drying oven (Yamato Scientific, Tokyo, Japan). Last, the α-TCP/CSs were sterilized using ethylene oxide gas at 40 °C. Collagen sponges prepared without α-TCP particles were prepared by the same method and used as a control [2].

### 4.2. Biomaterial Measurements

The morphologies of particles were observed by SEM (5-kV, S-4800, Hitachi High Technologies, Tokyo, Japan). FT-IR spectra were acquired using a spectrometer (Spectrum One, Perkin-Elmer Inc., Waltham, MA, USA) with ATR accessories at a resolution of 4 cm^−1^ with 16 scans. Sample identification was determined by XRD-6100 powder X-ray diffractometry (Shimadzu Corp., Kyoto, Japan). Cu-Kα radiation generated at 40 kV and 40 mA. The scan rate was 4°/min with a step size of 0.02° over a 2θ range of 10°–60°.

### 4.3. Rat Calvarial-Defect Model

A total of 28 male Sprague-Dawley rats (8 weeks old, 250–270 g, SHIMIZU Laboratory Supplies Co., Kyoto, Japan), which provided for 28 defect sites, were used for the implantation studies. The experimental protocol was approved by the Animal Care and Use Committee of Osaka Dental University (Admission Number: 14-05001). Three groups were prepared as follows: (i) no treatment control group (4-, 6-, 8-week groups; *n* = 4/group); (ii) Zol injection group (6- and 8-week groups; *n* = 4/group); and (iii) IFN-γ injection group (6- and 8-week groups; *n* = 4/group). A critically sized defect (diameter: 9 mm; depth: 1.0 mm) was created at the center of each rat skull. The α-TCP/CSs were implanted into defects and were covered using GTR membranes (Japan Gore-Tex Co., Tokyo, Japan). The operation was performed under strictly aseptic conditions. A pain reliever (Lepetan^®^, Otsuka Pharmaceutical, Tokyo, Japan) and a systemic antibiotic (Baytril^®^, Bayer, Leverkusen, Germany) were administered prophylactically by subcutaneous injection. Nine micrograms of IFN-γ (Bioss, Woburn, MA, USA; 4.5μg/mL) was locally and subcutaneously injected at the operation site (3 days/week for 4 weeks after the operation) and Zol-group rats receiving local and subcutaneous injection (0.04 mg/kg) of Zol (100 μg/mL; Cayman Chemical Company, Ann Arbor, MI, USA) at the operation site once a week. Rats were injected with calcein (5 mg/kg; Wako Pure Chemical Industries Co., Osaka, Japan) was injected 3 days/week from 4 to 6 weeks post-implantation. Tetracycline (25 mg/kg; Wako Pure Chemical Industries Co., Osaka, Japan) was administered 3 days/week from 6 to 8 weeks post-implantation.

### 4.4. Evaluation of Osteogenesis in Vivo

#### 4.4.1. Micro-CT and Analysis

Calvarial bones were scanned with an SMX-130CT micro-CT scanner (65 kV, 90 mA; Shimadzu, Kyoto, Japan) immediately after the rats were euthanized. Calvarial bones were measured in 3 dimensions, and their structural indices (voxel size: LX: 136; LY: 127 LZ 58) were calculated using a morphometric program (TRI/3D-BON; Ratoc System Engineering, Tokyo, Japan).

#### 4.4.2. RT-PCR and Immunostaining Experiments

Total RNA from each bone defect was extracted using the RNeasy Lipid Tissue Mini Kit (Qiagen, Hilden, Germany). RT-PCR was used to investigate the effects of injection on the expression of 5 genes, which were divided into 2 groups. Specifically, the genes were grouped as (i) those that are closely related to osteoclast differentiation and bone resorption (*Rankl*, *M-csf*, *Tnf-α*, and *Il-1β*) and (ii) one that is related to bone metabolism (*Alp*). Expression of *18S* mRNA was evaluated using a Gene Expression Assay (#4310893E, ThermoFisher Scientific Inc., Waltham, MA, USA). The sequences of the primers used for RT-PCR are shown in Table 1.

**Table 1:** Primers and probes for RT-PCR analysis.

Gene	Sequence		#Probe	Accession
*Rankl*	Forward	5ʹ-AGACACAGAAGCACTACCTGACTC-3ʹ	#2	NM_057149
	Reverse	5ʹ-GGCCCACAATGTGTTGTA-3ʹ		
*M-csf*	Forward	5ʹ-CAAGGACTATAAGGAACAGAACGAG-3ʹ	#55	NM_023981.4
	Reverse	5ʹ-GAAATTCTTGATTTTCTCCAGCA-3ʹ		
*Tnf-*α	Forward	5ʹ-GCCCAGACCCTCACACTC-3ʹ	#119	X66539.1
	Reverse	5ʹ-CCACTCCAGCTGCTCCTC-3ʹ		
*Il-1*β	Forward	5ʹ-TGTGATGAAAGACGGCACAC-3ʹ	#78	NM_031512.2
	Reverse	5ʹ-CTTCTTCTTTGGGTATTGTTTGG-3ʹ		
*Alp*	Forward	5ʹ-GCACAACATCAAGGACATCG-3ʹ	#77	NM_013059.1
	Reverse	5ʹ-TCAGTTCTGTTCTTGGGGTACAT-3ʹ		

Immunostaining was used to detect TNF-α and RANKL expression in bone-defect sections. Endogenous peroxidase and alkaline phosphatase activities were blocked using a blocking solution (BLOXALL, Vector; Burlingame, CA, USA) for 10 min. Goat serum (5%, Vector; Burlingame, CA, USA) was applied for 30 min to block background staining in the sections. Sections were labeled with one of the following primary antibodies diluted in PBS for 30 min: (a) an anti-TNF-α rabbit polyclonal antibody diluted at 1:300 (Novus Biologicals; Littleton, CO, USA); or (b) an anti-RANKL goat polyclonal antibody diluted at 1:300 (Santa Cruz Biotechnology, Dallas, TX, USA). After a 5-min wash step, the sections were incubated with an anti-rabbit or anti-goat secondary antibody (Vector, Burlingame, CA, USA) for 30 min. Subsequently, the sections were washed for 5 min and processed using the ABC Kit (Vector; Burlingame, CA, USA). Peroxidase activity was detected with the following colors: (a) purple with TNF-α staining (VECTOR VIP, Vector, Burlingame, CA, USA); (b) brown with RANKL staining (ImmPACT DAB, Vector, Burlingame, CA, USA). Counterstaining was performed using toluidine blue (RANKL staining) or methyl green (TNF-α staining).

#### 4.4.3. Bone Histomorphometry

Samples were fixed in 4% paraformaldehyde for 16 h. Four-micrometer-thick non-decalcified frozen sections were obtained by the Kawamoto method [29]. Osteogenesis dynamics were studied by observing fluorescently labeled sections from the 8-week group under an LSM700 laser-scanning microscope (Zeiss, Jena, Germany). The fluorophores were activated using lasers of different wavelengths, namely, 488 nm (calcein, blue) or 405 nm (tetracycline, green).

#### 4.4.4. Histochemical Staining and Histological Observations

Von Kossa, ALP, and TRAP staining were performed for histological studies. Von Kossa staining, using the von Kossa Method for Calcium Kit (Polysciences Inc., Warrington, UK), was performed for the bone tissue observations. TRAP and ALP staining was performed using the TRAP/ALP Kit (Wako Pure Chemical Industries Co., Osaka, Japan) for osteoclast identification and measuring osteoblast activities following transplantation. After staining, sections were observed with a BZ-9000 digital microscope (Keyence Co., Osaka, Japan).

### 4.5. Statistical Analysis

Statistical analysis was performed with Statcel3 software (OMS, Tokyo, Japan). For all experiments, values are reported as the mean ± SD. For comparisons between 3 groups, homogeneities of variance were evaluated by the Bartlett test. Since all data were homoscedastic, differences were evaluated by Tukey–Kramer method (a multiple-comparisons test for parametric data). A value of *p* < 0.05 was accepted as statistically significant.

## 5. Conclusions

Although α-TCP/CSs are osteoconductive materials that act as a space maintainer during bone formation, combination treatment with molecules that stimulate osteogenesis or inhibit osteoclastogenesis are needed to induce bone regeneration in large bone defects. In this study, we demonstrated that locally Zol- or IFN-γ-injected bone defects with α-TCP/CS implants exhibited significantly higher bone regeneration and decreased inflammatory cytokine expression, leading to a reduction in osteoclastogenesis in new bones. These data indicate that Zol and IFN-γ may have utility in mediating successful material implantation. Further studies are required to examine the therapeutic potential of Zol and IFN-γ administration, using different implant procedures and types.
